# Therapeutic targeting of p300/CBP HAT domain for the treatment of NUT midline carcinoma

**DOI:** 10.1038/s41388-020-1301-9

**Published:** 2020-05-04

**Authors:** Xin Zhang, Tim Zegar, Anais Lucas, Chevaun Morrison-Smith, Tatiana Knox, Christopher A. French, Stefan Knapp, Susanne Müller, Jens T. Siveke

**Affiliations:** 1 Institute for Developmental Cancer Therapeutics, West German Cancer Center, University Hospital Essen, Essen, Germany; 2 Division of Solid Tumor Translational Oncology, German Cancer Consortium (DKTK, Partner Site Essen) and German Cancer Research Center, DKFZ, Heidelberg, Germany; 3 Structural Genomics Consortium, Buchmann Institute for Life Sciences, Goethe University Frankfurt, 60438 Frankfurt, Germany; 4 Institute of Pharmaceutical Chemistry, Goethe University Frankfurt, 60438 Frankfurt, Germany; 5 German Cancer Consortium (DKTK partner site Frankfurt/Mainz) and Frankfurt Cancer Institute (FCI), 60438 Frankfurt, Germany; 6 Brigham and Women’s Hospital/Harvard Medical School, Boston, MA USA

**Keywords:** Targeted therapies, Target identification

## Abstract

Nuclear protein of the testis (NUT) midline carcinoma (NMC), is a rare and highly aggressive form of undifferentiated squamous cell carcinoma. NMC is molecularly characterized by chromosomal rearrangement of the *NUT* gene to another gene, most commonly the bromodomain and extraterminal domain (BET) gene *BRD4*, forming the *BRD4-NUT* fusion oncogene. Therefore, inhibiting BRD4-NUT oncogenic function directly by BET inhibitors represents an attractive therapeutic approach but toxicity may limit the use of pan-BET inhibitors treating this cancer. We thus performed a drug screening approach using a library consisting of epigenetic compounds and ‘Donated Chemical Probes’ collated by the Structural Genomics Consortium (SGC) and identified the p300/CBP HAT inhibitor A-485, in addition to the well-known BET inhibitor JQ1, to be the most active candidate for NMC treatment. In contrast to JQ1, A-485 was selectively potent in NMC compared to other cell lines tested. Mechanistically, A-485 inhibited p300-mediated histone acetylation, leading to disruption of BRD4-NUT binding to hyperacetylated megadomains. Consistently, BRD4-NUT megadomain-associated genes *MYC*, *CCAT1* and *TP63* were downregulated by A-485. A-485 strongly induced squamous differentiation, cell cycle arrest and apoptosis. Combined inhibition of p300/CBP and BET showed synergistic effects. In summary, we identified the p300/CBP HAT domain as a putative therapeutic target in highly therapy-resistant NMC.

## Introduction

Nuclear protein of the testis (NUT) midline carcinoma (NMC) is defined by chromosomal rearrangement of the nuclear protein of the testis (*NUT*) gene on chromosome 15q14 mainly arising in midline structures, such as head, neck and mediastinum. In ~70% of NMCs, most of the coding sequence of NUT is fused to BRD4, creating a *BRD4-NUT* oncogene [1, 2]. In the BRD4-NUT fusion protein, the BRD4 moiety contains two tandem bromodomains (BD) that bind to acetyl-lysine residues on histones and the NUT moiety contains two acidic domains (AD), one of which binds to the histone acetyltransferase p300/CBP stimulating its catalytic activity [3]. Recruitment of p300/CBP leads to regional histone hyperacetylation, which further recruits BRD4-NUT in a feed-forward manner [4]. Eventually, massive acetylated chromatin regions termed ‘megadomains’ are created. BRD4-NUT megadomains drive transcription of underlying genes (e.g. *MYC* and *TP63*) that prevent differentiation and stimulate growth [4].

NMC is one of the most therapy-resistant tumors. As a major pathogenic driver of transformation, BRD4-NUT represents a rationale target for NMC. In preclinical models, BET inhibitors (BETi) that compete with acetyl-lysines on histones for binding of BRD4 have shown anti-proliferative efficacy accompanied with squamous differentiation [5]. Exposure of NMC cells to BETi results in the loss of BRD4-NUT megadomain and downregulation of megadomain*-*associated genes [4]. Several BETi have entered clinical trials and evidence of clinical activity was observed [6–8]. However, the response rate in NMC to BETi was only 20–30% and patients eventually developed resistance [6, 7]. Another concerning issue is toxicity of pan-BETi, leading most commonly to thrombocytopenia, thus limiting the usage of BETi in NMC [6, 7]. Therefore, alternative regimens or combination therapies need to be developed. In this study, we identified a p300/CBP HAT inhibitor that is selectively potent in NMC. Consistent with the location of p300/CBP in a complex with BRD4-NUT, this inhibitor disrupts BRD4-NUT megadomain and downregulates megadomain-associated genes, leading to squamous differentiation and growth arrest. Additionally, p300/CBP and BET inhibitors confer synergistic anti-tumor effects. These results implicate an alternative regimen in NMC by targeting p300/CBP as a monotherapy or combined with BETi.

## Results

### A-485 is selectively anti-proliferative in NMC

In order to identify potential inhibitors for NMC, we screened two libraries of highly selective and well-characterized inhibitors, so called chemical probes that have been developed by the Structural Genomics Consortium (SGC) chemical probe program (epigenetic library) or have been donated by industry (donated chemical probes, DCP). Each compound of these libraries is accompanied by its inactive structurally highly related analogues. The potency, selectivity and cellular activity of all the compounds have been extensively profiled and data are regularly updated in an online database and web resources (https://www.sgc-ffm.uni-frankfurt.de/ and https://www.thesgc.org/chemical-probes/epigenetics). The currently assembled 79 chemical probes cover diverse cellular targets such as epigenetic regulators, receptors and transporters as well as kinases [9–11] (Supplementary Fig. 1).

**Fig. 1:**
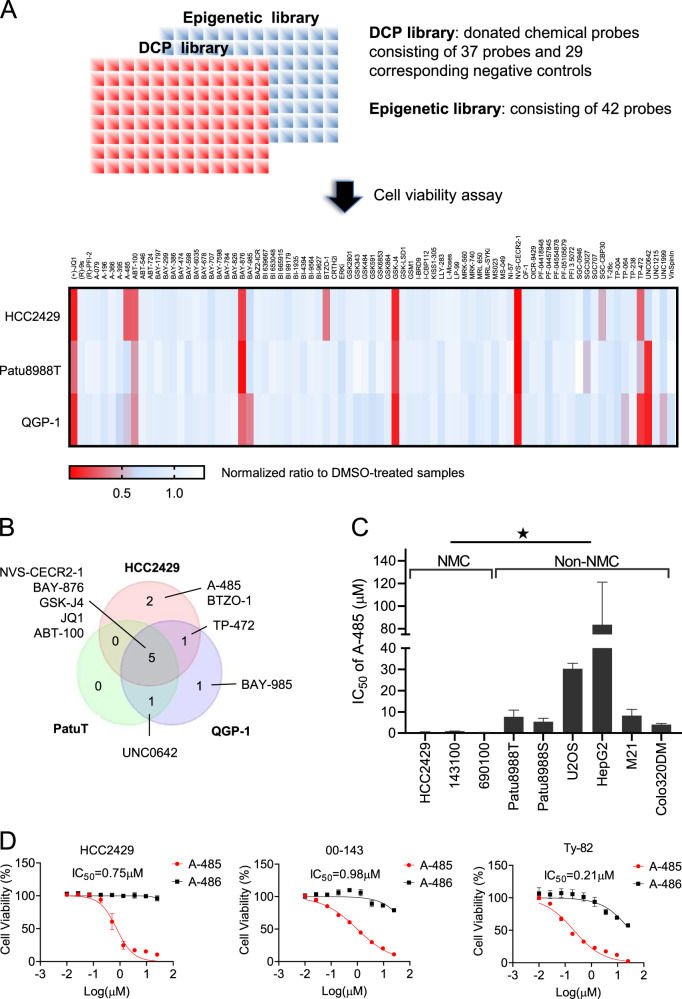
Chemical probe screening identified a p300/CBP inhibitor that is selectively anti-proliferative in NMC. **a** Chemical probe screening in three tumor cell lines. HCC2429, NUT midline carcinoma; Patu8988T, pancreatic ductal adenocarcinoma; QGP-1, pancreatic neuroendocrine tumor. Cells were incubated with each of the chemical probes at a concentration of 10 µM for 72 h and cell viabilities were measured by CellTiter Glo Cell Viability assay. The values were normalized to dimethyl sulfoxide (DMSO)-treated samples and a heatmap was generated based on the mean values of three independent experiments. The heat map was colored according to normalized cell viability as depicted in the figure capture. The *p*-values of positive hits (JQ1, A-485 and BTOZ-1) were presented in the text. **b** Venn diagram analysis showing NMC-selective and -unselective inhibitors. Probes with cell viability less than 50% in at least one cell line from the screening above were chosen as potent hits. **c** IC_50_ of A-485 on three NMC cell lines and six cell lines of other tumor identities. Mean ± SEM from three independent experiments, *P ≤ 0.05. **d** Comparison of the growth effects A-485 (red circles) and the inactive analogue A-486 (black square) on three NMC cell lines. IC_50_ of A-485 is shown in the graph. Mean ± SD from three technical replicates. In (**c**) and (**d**), cells were incubated with inhibitors at a concentration range between 10 nM and 25 µM. Cell viability was monitored after 72 h by CellTiter Glo Cell Viability assay. The dose response curve was used to determine the IC50 by Prism.

Next, we analyzed the NMC cell line HCC2429 and two pancreatic tumor cell lines (Patu8988T and QGP-1) to identify NMC-selective inhibitors and distinguish them from compounds of general toxicity (Fig. 1a). Among the chemical probes that showed strong anti-proliferative activity in HCC2429 cells, we identified the BETi JQ1 (*P* < 0.0001; Fig. 1a, b). Consistent with previous studies [12], JQ1 also showed strong activity against non-NMC cells (Fig. 1a, b). In contrast, two compounds, A-485 (*P* = 0.016) and BTZO-1 (*P* = 0.028), only showed activities in HCC2429 cells (Fig. 1a, b). A-485 was developed as a selective catalytic p300/CBP inhibitor, which has demonstrated inhibitory effects in several hematological malignancies and androgen receptor-positive prostate cancer [13]. BTZO-1, a selective inhibitor for macrophage migration inhibitory factor (MIF), was originally discovered as a cardioprotective agent [14]. However, the role of MIF in NMC remains to be established and this strategy was not pursued further in this study.

Considering the important roles of p300/CBP in NMC, we focused on A-485 for further characterization. To validate our findings, we determined the half maximal inhibitory concentration (IC_50_) values of A-485 across three NMC cell lines (HCC2429, 00–143 and Ty-82) and six non-NMC cell lines (Patu8988T, Patu8988S, U2OS, HepG2, M21 and COLO320DM). We found a significantly higher activity in all NMC cell lines compared to non-NMC cells (Fig. 1c). Supporting an on-target action of A-485, the inactive analogue A-486 yielded no activity in NMC cells (Fig. 1d).

### A-485 impairs hyperacetylated chromatin domains and downregulates BRD4-NUT megadomain-associated genes

**Fig. 2:**
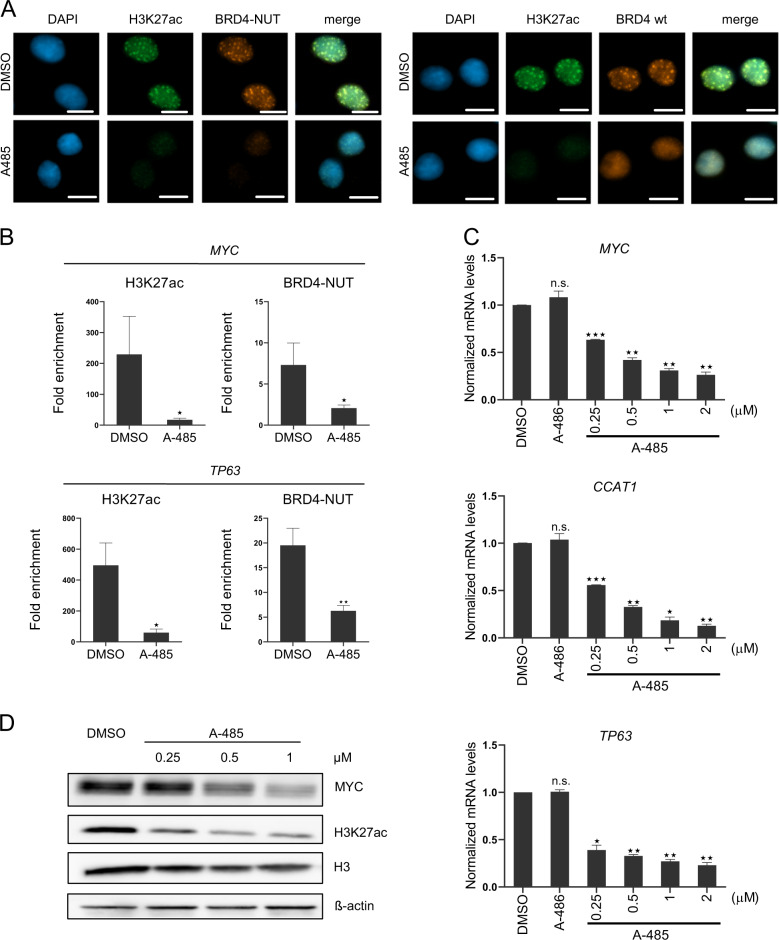
A-485 impairs hyperacetylated chromatin domains and downregulates BRD4-NUT megadomain-associated genes. **a** Immunofluoresence detection of H3K27ac, BRD4-NUT and BRD4 wt proteins in HCC2429 cells incubated with 1 µM A-485 or DMSO for 3 days. Scale bar = 10 µm. **b** Chromatin immunoprecipitation (ChIP) analysis of H3K27ac and BRD4-NUT at the *MYC* promoter and *TP63* enhancer regions in HCC2429 cells incubated with 1 µM A-485 or DMSO for 3 days. Chromatin was precipitated with normal rabbit IgG (IgG as control), H3K27ac and NUT antibodies. Precipitated chromatin was analyzed using qPCR and presented as fold enrichment to IgG control. Mean ± SEM from four independent experiments, **P ≤ 0.01, *P ≤ 0.05. **c** Quantitative RT-PCR analysis of *MYC*, *CCAT1* and *TP63* genes and (**d**) immunoblot analysis of H3K27ac and MYC proteins in HCC2429 cells incubated with A-485 at indicated concentrations for 48 h. Mean ± SEM from three independent experiments, ***P ≤ 0.001, **P ≤ 0.01, *P ≤ 0.05; n.s., not significant.

Because of the critical roles of p300/CBP in creating hyperacetylated chromatin domains associated with BRD4-NUT in NMC [3, 4], we explored the consequences of p300/CBP inhibition by A-485. First, we performed immunofluorescence analysis in HCC2429 cells. In DMSO-treated cells, BRD4-NUT and BRD4 expressed from wild-type allele (BRD4 wt) were co-localized with acetylated H3K27 (H3K27ac) in the distinct chromatin foci (Fig. 2a). A-485 treatment dispersed the hyperacetylated chromatin foci (Fig. 2a). Similar effects were observed in NMC cell lines TC-797 and PER-403 (Supplementary Fig. 2A). We further observed that BRD4-NUT and BRD4 wt protein levels were decreased by A-485 (Supplementary Fig. 2B).

Previous studies demonstrated that BRD4-NUT megadomains overlap at oncogenic loci and induce abnormal expression of oncogenes (e.g. *MYC, CCAT1* and *TP63*) in NMC [4]. *CCAT1* is an enhancer RNA upstream of *MYC* locus [15], and *CCAT1* and *MYC* share one BRD4-NUT megadomain [4]. We assumed that p300/CBP inhibition could impair BRD4-NUT binding at these oncogenic loci due to the diminished acetylated histone. To confirm this, we performed chromatin immunoprecipitation. Indeed, we observed diminished H3K27ac and BRD4-NUT levels at the *MYC* promoter and *TP63* enhancer regions in A-485-treated HCC2429 cells (Fig. 2b). Consistently, *MYC, CCAT1* and *TP63* mRNA levels were significantly repressed by A-485 at a very early time point (6 h, Fig. 2c), suggesting a direct effect of A-485 on the expression of these genes. Similar effects were observed in TC-797 and PER-403 cells (Supplementary Fig. 3A). MYC protein levels were also reduced in A-485-treated HCC2429 cells (Fig. 2d).

To further elucidate the specific role of A-485 on p300/CBP, we performed *p300*/*CBP* loss-of-function experiment. The siRNAs showed moderate repression of *p300* and *CBP* mRNA levels respectively (Supplementary Fig. 3B). Since A-485 targets the HAT domain of both p300 and CBP, we combined *p300* and *CBP* siRNAs for the knockdown experiment to maximally phenocopy A-485. In agreement with A-485, double knockdown of *p300*/*CBP* also downregulated *MYC, CCAT1* and *TP63* mRNA levels supporting target-specific effects of A-485 (Supplementary Fig. 3C). These results indicate that p300/CBP inhibition by A-485 efficiently impairs BRD4-NUT oncogenic functions in NMC.

### A-485 induces squamous differentiation, cell cycle arrest and apoptosis

**Fig. 3:**
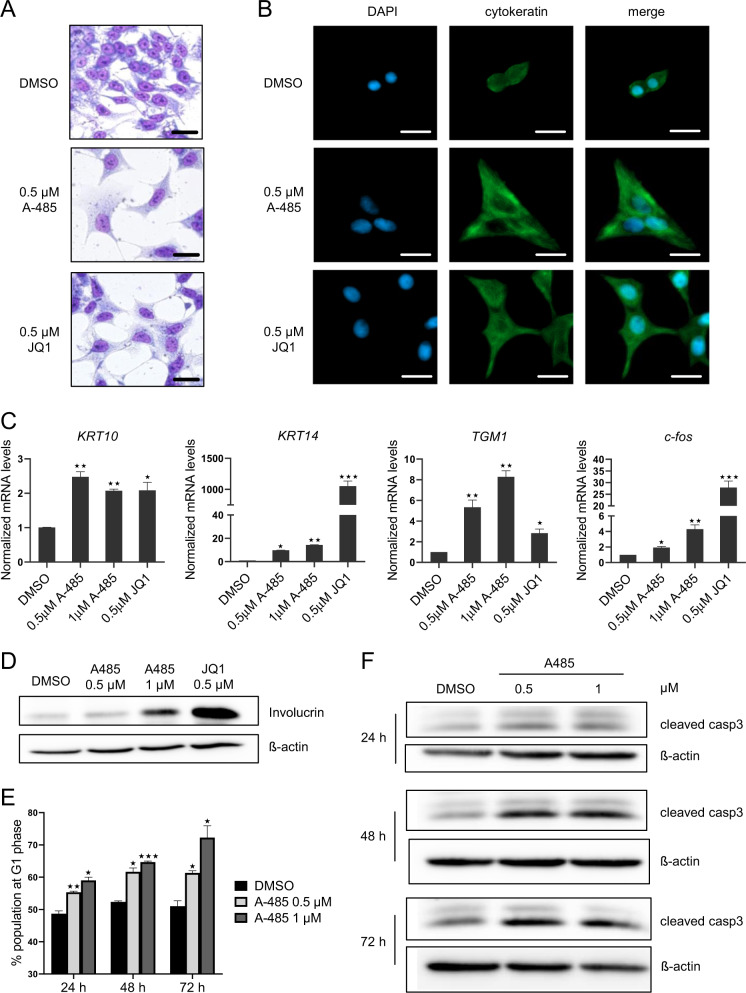
A-485 induces squamous differentiation, cell cycle arrest and apoptosis. **a** Hemacolor staining of HCC2429 cells incubated with 0.5 or 1 µM A-485 for 5 days. **b** Immunofluoresence detection of cytokeratin in HCC2429 cells incubated with 0.5 µM A-485 or JQ1 for 5 days. Scale bar = 20 µm. **c** Quantitative RT-PCR analysis of squamous tissue genes (*KRT10*, *KRT14* and *TGM1*) and *c-fos* in HCC2429 cells incubated with 0.5 or 1 µM A-485 or 0.5 µM JQ1 for 5 days. Mean ± SEM from three independent experiments, ***P ≤ 0.001, **P ≤ 0.01, *P ≤ 0.05. **d** Immunoblot analysis of Involucrin in HCC2429 cells incubated with 0.5 or 1 µM A-485 or 0.5 µM JQ1 for 5 days. **e** Flow cytometry analysis of HCC2429 cells incubated with 0.5 or 1 µM A-485 for 24, 48 and 72 h. Mean ± SEM from three independent experiments, ***P ≤ 0.001, **P ≤ 0.01, *P ≤ 0.05. **f** Immunoblot analysis of cleaved caspase-3 in HCC2429 cells incubated with 0.5 or 1 µM A-485 for 24, 48 and 72 h.

We reasoned that if competitive inhibition of BRD4-NUT in NMC is sufficient to induce squamous differentiation [5], A-485 might also provoke differentiation by disrupting BRD4-NUT megadomains. Indeed, A-485-treated HCC2429 cells showed a differentiation phenotype, featured by flattening of cells and accumulation of pan-keratin in the cytoplasm (Fig. 3a, b). Expression analysis by quantitative RT-PCR showed induction of three canonical squamous tissue genes (*KRT10*, *KRT14* and *TGM1*) in a dose-dependent manner (Fig. 3c). *C-fos*, belonging to the Activation Protein-1 (AP-1) family, is an immediate-early inducible transcription factor required for normal epithelial cell differentiation [16]. Here, we also observed the induction of *c-fos* by A-485 (Fig. 3c). Furthermore, A-485 induced the protein levels of Involucrin, a well-known differentiation marker (Fig. 3d). Differentiation phenotype was also observed in TC-797 and PER-403 cells treated with A-485 indicated by morphological changes (Supplementary Fig. 4A). Although TC-797 and PER-403 have different cells of origin and varying degrees of capacity to differentiate, their marker profiles are in most consistent with that of HCC2429 cells (Supplementary Fig. 4B, C). Consistently, *p300*/*CBP* double knockdown in HCC2429 cells also induced *c-fos* expression (Supplementary Fig. 4D), although the induction of squamous tissue genes (*KRT10*, *KRT14* and *TGM1*) was not obvious probably due to the moderate downregulation of *p300*/*CBP* by siRNAs (Supplementary Fig. 3B). By performing chromatin immunoprecipitation analysis at the *c-fos* promoter region, we also observed diminished H3K27ac and BRD4-NUT enrichment upon A-485 treatment (Supplementary Fig. 5). It would be interesting to further dissect the mechanism of de-repression of differentiation gene by A-485.

In NMC cells, differentiation was shown to be accompanied by cell cycle arrest [5]. Indeed, A-485 induced G1 arrest in HCC2429 cells at early time point (24 h, Fig. 3e). Moreover, elevated levels of cleaved caspase-3 at later time points (48 and 72 h) indicated apoptosis induction by A-485 (Fig. 3f).

### P300/CBP and BET inhibition have synergistic effects in NMC

**Fig. 4:**
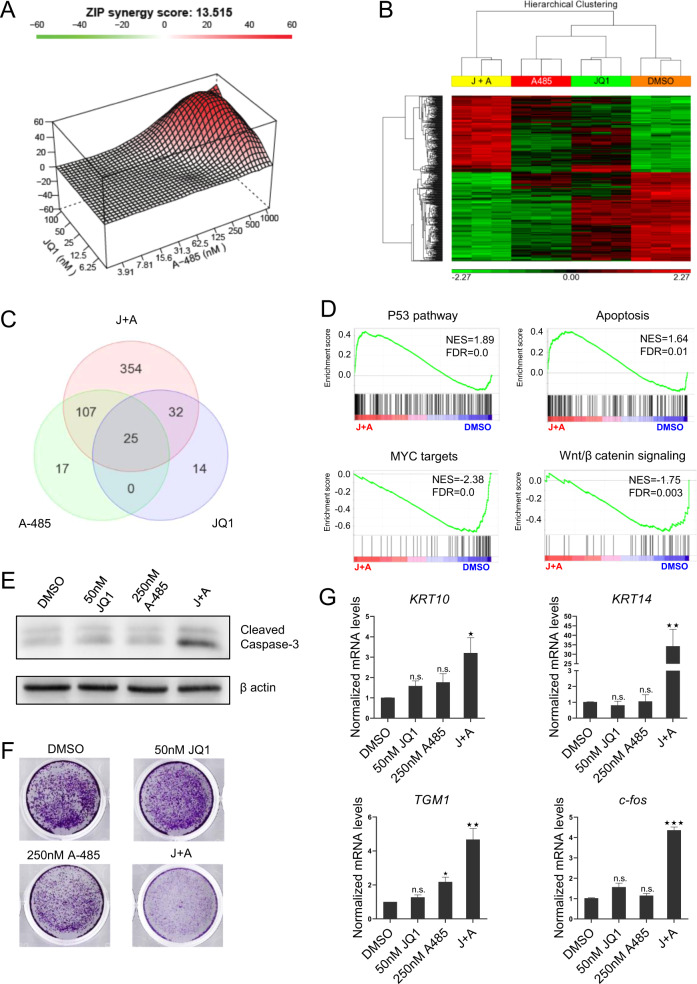
P300/CBP and BET inhibition have synergistic effects in NMC. **a** Combination response to A-485 and JQ1 for HCC2429 cells. CellTiterGlo cell viability assay was performed to measure cell viabilities of all the indicated dose combinations for 72 h. Synergy effects were evaluated using SynergyFinder (https://synergyfinder.fimm.fi). The ZIP synergy score is averaged over all the dose combination cells. **b**,**c** Hierarchical clustering (**b**) and Venn diagram analysis (**c**) of the differentially expressed genes in HCC2429 cells treated with 50 nM JQ1 and 250 nM A-485 alone or combined for 8 h. Each treatment was done in triplicate. **d** Representative GSEA plots showing significantly enriched up- and downregulated pathways (combination-treatment versus DMSO). **e** Immunoblot analysis of cleaved caspase-3 in HCC2429 cells incubated with 50 nM JQ1 and 250 nM A-485 alone or combined for 72 h. **f** Colony formation assay for HCC2429 cells incubated with 50 nM JQ1 and 250 nM A-485 alone or combined for 72 h. **g** Quantitative RT-PCR analysis of squamous tissue genes (*KRT10*, *KRT14* and *TGM1*) and *c-fos* in HCC2429 cells incubated with 50 nM JQ1 and 250 nM A-485 alone or combined for 5 days. Mean ± SEM from three independent experiments, ***P ≤ 0.001, **P ≤ 0.01, *P ≤ 0.05; n.s., not significant.

Because P300/CBP and BRD4-NUT co-localize in hyperacetylated chromatin foci in NMC, we assessed if combination of p300/CBP and BET inhibitors would lead to synergistic anti-proliferative effects. We tested 9 different concentrations of A-485 ranging from 3.91 nM to 1 µM in combination with 5 different concentrations of JQ1 ranging from 6.25 to 100 nM for HCC2429 cells. After 72 h incubation, cell viability assays were performed and the synergistic effects were evaluated using SynergyFinder [17]. Combined treatment of A-485 and JQ1 showed strong synergy (ZIP synergy score 13.514, Fig. 4a). We also tested combined treatment in a non-NMC cell line Patu8988S and still observed an albeit smaller synergistic effect (ZIP synergy score 7.531, Supplementary Fig. 6), arguing that combined inhibition of p300/CBP and BET may be synergistic beyond NMC cells.

To further explore this synergistic effect, transcriptomic profiling was performed in HCC2429 cells incubated with A-485 and JQ1 alone or combined at concentrations of 1/3 of cellular IC_50_ values for 8 h to evaluate the primary transcriptional effect of the inhibitors. Only 149 and 71 genes were affected by A-485 and JQ1 respectively (Fig. 4b, c), but combined treatment differentially regulated more genes (518 genes, Fig. 4b, c). To obtain insight into the gene expression patterns, we performed gene set enrichment analysis (GSEA). In combination-treated samples, the p53 pathway and apoptosis were among the most significantly enriched pathways (Fig. 4d), which probably contribute to the observed synergistic effects. Furthermore, gene sets for MYC targets and Wnt/β catenin signaling that support tumor cell growth and inhibit differentiation were significantly downregulated (Fig. 4d). Validating the above findings, immunoblot analysis showed enhanced cleaved caspase-3 by combined treatment (Fig. 4e), indicating induced apoptosis. Consistently, combined treatment, but not single treatment with sub-optimal concentrations, strongly inhibited colony formation (Fig. 4f and Supplementary Fig. 7A). Moreover, at concentrations below the IC_50_ values for the single agents, only combined treatment induced squamous differentiation (Fig. 4g and Supplementary Fig. 7B).

## Discussion

We identified the p300/CBP HAT inhibitor A-485 to be highly potent in NMC but not in tested cell lines derived from other tumor entities. Our chemical probe library also included two p300/CBP bromodomain inhibitors (I-CBP112 and SGC-CBP30) [18, 19]. However, both p300/CBP bromodomain inhibitors showed no or only marginally inhibitory effects on NMC cells raising the question if the bromodomain of p300/CBP is dispensable for its oncogenic function in NMC. In general, the bromodomain is required for p300/CBP to serve as acetyl-lysine binding module tethering the HAT activity to defined chromatin sites to achieve highly specific histone acetylation and transcriptional activation [20, 21]. In NMC, BRD4-NUT binds to acetylated chromatin through its bromodomains and provides a platform for the recruitment of p300/CBP and the stimulation of its HAT activity [3]. Moreover, the bromodomain of p300/CBP is not required for the direct interaction between p300/CBP and BRD4-NUT [3]. Therefore, we reasoned that the bromodomain might be dispensable for chromatin binding of p300/CBP in NMC. However, whether the bromodomain affects p300/CBP HAT activities in NMC is unknown. Further work will be required to compare the effects of the p300/CBP HAT and bromodomain inhibition to develop the most potent p300/CBP inhibitors.

NMC, one of the most lethal solid tumors, responds poorly to chemo- and radiotherapy. Since the discovery of BET proteins in the tumorigenesis of NMC, current efforts focus on targeting the causative oncoprotein BET. The main targets of the pan-BETi developed so far include BRD2, BRD3 and BRD4, which are ubiquitously expressed in tissues. Given the importance of BET proteins in the basal transcription machinery, BETi inevitably affect normal cell functions. Thrombocytopenia, fatigue, gastrointestinal symptoms, and hyperbilirubinemia are among the dose-limiting side effects reported in patients treated with BETi [7]. Pan-BETi was also reported to have activity for bromodomain testis-specific protein (BRDT), causing testicular atrophy and reversible infertility [22]. Compared to the activity of BETi across broad tumor types, p300/CBP inhibitors selectively target lineage-specific tumors [13]. Moreover, transcriptional profiling of human T cells and one prostate cancer cell line after treatment of p300/CBP inhibitors revealed significantly fewer altered genes than observed with BETi [19, 23]. Thus, p300/CBP inhibition is an alternative therapeutic strategy that potentially leads to fewer adverse events than the broadly acting BETi.

In clinical trials of BEiT, only a small fraction of NMC patients responded and eventually relapsed during treatment [6, 7]. Thus, the development of BETi faces the challenges of how to enhance the sensitivity of patients and how to overcome resistance. Others [18, 23] and our study discovered that combination of p300/CBP and BETi results in a highly synergistic inhibitory effect in several tumor types. Furthermore, BETi-resistant cells continue to respond to the p300/CBP inhibitor [23]. We propose that combination therapy using both p300/CBP and BET inhibitors may be necessary to sensitize patient and overcome BETi resistance. Our efforts in exploring the molecular mechanisms of this synergistic effect in NMC discovered that combined p300/CBP and BET inhibitors significantly downregulate Wnt/β catenin signaling. Interestingly, one study in human and mouse leukemia cells demonstrated that increased Wnt/β catenin signaling contributes to the resistance to BETi and negative regulation of this pathway restores the sensitivity [24]. Recently, a dual inhibitor of both p300/CBP and BET showed promising anti-tumor effect in prostate cancer [25, 26]. Thus, combined p300/CBP and BET inhibition may be a rational and conceivable targeting approach in NMC and other tumor types.

## Materials and Methods

### Cell culture

NMC cell lines HCC2429 [27], Ty-82 [28], 00–143 [29], TC-797 [30], PER-403 [31] and the pancreatic tumor cell line QGP-1 [32] have been described. HCC2429, Ty-82 and 00–143 were kindly provided from Lead Discovery Center GmbH (Dortmund, Germany). The pancreatic tumor cell line Patu8988T was from the American Type Culture Collection. All cell lines were free of mycoplasma contamination and authenticated using short tandem repeat (STR) profiling.

## Supplementary information

Supplementary information

## Data Availability

Microarray data are available through ArrayExpress under the accession code E-MTAB-8955.
