# Supplementary material for: Therapeutic targeting of p300/CBP HAT domain for the treatment of NUT midline carcinoma
Source: Oncogene. 2020 May 4;39(24):4770–9. doi: 10.1038/s41388-020-1301-9 (PMC7286816; doi:10.1038/s41388-020-1301-9)

SUPPLEMENTARY INFORMATION

Extended Materials and methods

Cell culture and reagents

HCC2429 was cultured in RPMI1640 medium containing 10% FBS, 2 mM L-glutamine and 1% penicillin-streptomycin. Ty-82, 00-143 and TC-797 were cultured in Dulbecco's Modified Eagle Medium (DMEM) containing 10% FBS, 2 mM glutamine and 1% penicillin-streptomycin. PER-403 was cultured in the same media with 1 mM sodium pyruvate, 0.1 mM non-essential amino acids and 40 µM β–mercaptoethanol. Patu8988T and QGP-1 were cultured in DMEM containing 10% FBS, 25 mM glucose, 4 mM L-glutamine, 1 mM sodium pyruvate and 1% penicillin-streptomycin. Chemical probes were collected and donated by Structural Genomics Consortium (SGC). JQ1 was purchased from Biomol and dissolved in DMSO as 10 mM stock.

Screening

At day 1, cells were seeded in 96-well plates (HCC2429, 7000 cells/well; Patu8988T, 2000 cells/well; QGP-1, 5000 cells/well). Day 2, chemical probes were added to the wells at a working concentration of 10 µM. Day 5, cell viabilities were measured by CellTiterGlo Cell Viability assay. The cell viability values were normalized to DMSO-treated samples. Probes with cell viability less than 50% in at least one cell line from the screening above were chosen as potent hits.

RNA extraction and quantitative RT-PCR analysis

Total RNA was isolated using Maxwell® RSC simplyRNA Cells Kit (Promega). To detect normal gene expression, the isolated RNA was reverse-transcribed using PrimeScript Reverse Transcriptase (TaKaRa). The relative mRNA levels were determined by quantitative PCR with LightCycler ®480 SYBR Green I Master (Roche). PCR conditions were 5 min at 95°C, followed by 45 cycles of 95°C for 10 s, 59°C for 10 s and 72 °C for 20 s. The mRNA expression levels were normalized to GUSB or GAPDH and calculated using the 2- ΔΔCt method. The primer sequences are given in the supplementary Table S1.

CellTiterGlo cell viability assay

The assay was performed according to the manufacturer's instructions (Promega, G7571). Briefly, cells were seeded in drug-printed 96-well plates (Corning). 72 h later, one volume of diluted CellTiterGlo Reagent (1:4 with PBS) was added to the wells. Plates were shaken for 2 min and incubated for another 10 min. Luminescent signals were read by the Spark Multimode Microplate Reader (Tecan). The values of luminescent signals were normalized to the DMSO control wells and presented as percentage of cell viability. IC50 was calculated by GraphPad Prism 8.

Immunofluorescence

Cells were washed with PBS and fixed with 4 % PFA in PBS for 15 minutes. After washing with PBS again, cells were permeated with 0.3 % TritonX-100 in PBS for 10 minutes. A solution of 5 % BSA in PBS was used for blocking for 45 minutes at room temperature. Primary antibodies were dissolved in 1 % BSA in PBS and cells were incubated at 4° C overnight. The following secondary antibodies were used: goat anti-rabbit IgG (H+L) Cross-Adsorbed Secondary Antibody, DyLight 550 (SA5-10033, ThermoFisher Scientific) and goat anti-mouse IgG (H+L) Cross-Adsorbed Secondary Antibody, DyLight 488 (35503, ThermoFisher Scientific). Afterwards, slides were washed with PBS and counterstained with DAPI (Vectashield, H-1200, Vector Laboratories). The following primary antibodies were used: mouse anti H3K27ac (39685, Active Motif), rabbit anti NUT (3625, Cell Signaling Technology), rabbit anti BRD4-recombinant monoclonal (A700-004, Bethyl Laboratories), mouse anti pan-cytokeratin (ab6401, Abcam). Pictures were taken at a magnification of 40x.

Immunoblot analysis

Cells were lysed in RIPA buffer (9806S, Cell Signaling Technology) containing protease inhibitors (Roche). Protein extracts were separated on SDS-polyacrylamide gels, transferred to nitrocellulose membranes with Trans-Blot® Turbo™ Transfer System (Bio-Rad) and incubated with antibodies dissolved in TBS containing 5% BSA and Tween 20 (0.1%). The following primary antibodies were used: rabbit anti-c-myc, (9402, Cell Signaling Technology), rabbit anti-ß-actin (ab8227, Abcam), rabbit anti-cleaved caspase-3(Asp175) (5A1E, Cell Signaling Technology), rabbit anti Acetyl-Histone H3 (Lys27) (8173, Cell Signaling Technology), rabbit anti-NUT(C52B1) (3625, CST), mouse anti-involucrin (I9018, Sigma-Aldrich), rabbit anti-BRD4[EPR5150(2)] (ab128874, Abcam), rabbit anti-Histone H3 (ab1791, Abcam). Primary antibodies were detected by a peroxidase-coupled secondary antibody (Jackson) and chemiluminescence (ThermoFisher).

Cell cycle analysis

The attached and floating cells were harvested and fixed in 70% ethanol at 4°C overnight. Cells were then washed, rehydrated and resuspended in PBS containing 0.5 mg/ml RNase A. Cells were incubated at 37°C for 30 min and then 30 µg/mL propidium iodide was added. The cellular DNA contents were analyzed by the Guava Easycyto System.

Chromatin immunoprecipitation (ChIP)

Cells were seeded in 15 cm culture dishes, and treated with inhibitors for indicated time period. Chromatin immunoprecipitation was performed using SimpleChIP Enzymatic Chromatin IP Kit (9003,

Cell Signaling Technology). The following antibodies were used for ChIP: rabbit anti Acetyl-Histone H3 (Lys27) (8173, Cell Signaling Technology), rabbit anti NUT (3625, Cell Signaling Technology), and normal rabbit IgG (2729, Cell Signaling Technology). The primers used for ChIP qPCR are given in the Supplementary Table S1. Data presented as fold enrichment to IgG control.

siRNA transfection

P300 and *CBP* siRNAs were purchased from Thermo Fisher Scientific (Catalogue Nr. 4427037-s4696 and 4427038-s3495, respectively). siRNA transfection was performed using Lipofectamine 2000 according to manufacturer's instructions (11668019, Thermo Fisher Scientific).

Hemacolor staining

Cells were washed once with PBS and air-dried. Hemacolor staining was performed according to manufacturer's instructions (1116610001, Millipore Sigma).

Global gene expression profiles

Microarray analysis using HumanHT-12 v4 Expression BeadChip (Illumina, Inc., San Diego, CA, USA) was carried out by the Genomics & Proteomics Core Facility at the German Cancer Research Center (Heidelberg, Germany) following the manufacturer's instructions. Raw data were normalized based on the quantile method. All data analysis and visualization of differentially expressed genes were conducted using Partek Genomics Suite 7.0. Gene set enrichment analysis (GSEA) was conducted using default settings on mean expression values from microarray data [1]. Sets included in the analysis ranged from 30 to 2000 genes, and gene set was used as a permutation type.

Statistical analysis

GraphPad Prism 8 was used for statistical analysis and visualization. Unpaired Mann-Whitney test was performed for statistical comparison of IC_{50} values between NMC and non-NMC cell lines. Paired, two-tailed parametric *t*-test was performed for all other experiments. Data for at least three independent experiments are expressed as mean ± SEM (standard error of the mean) or Mean ± SD (standard deviation). *P*-values < 0.05 was considered to be statistically significant.

Supplementary Table S1. Primer sequences.

qPCR primers for gene expression analysis

Gene	Forward sequence	Reverse sequence	Ref.
MYC	CAGCTGCTTAGACGCTGGATT	GTAGAAATACGGCTGCACCGA	[2]
CCAT1	CATTGGGAAAGGTGCCGAGA	ACGCTTAGCCATACAGAGCC	[3]
TP63	TGGAAACCAGAGATGGGCAA	CGGGCGCTTCGTACCATC	[4]
KRT10	AGGGGGCAGTTTCGGAGGTG	AAGTAGGAAGCCAGGCGGTCATT	[5]
KRT14	CCAGTTCTCCTCTGGATCGCAG	GATCTTCCAGTGGGATCTGTGTCCA	[6]
TGM1	ACATGAAGTACGACACGCCT	TTGGAGCTGATGGCCTTTGT	
c-fos	TGCCTCTCCTCAATGACCCTGA	ATAGGTCCATGTCTGGCACGGA	[7]
P300	AACAGCAGCTCAACCATCCA	TCCGGCGTAGGAAATATGGC	[8]
CBP	ACCGGTGTAAGGAAAGGCTG	TCAGGTGTTGGGAAGATGGC	[8]

ChIP primers

Gene	Forward sequence	Reverse sequence	Ref.
TP63	AGCCTGGCTCTTTAGTTGCC	CTAGCAGGGAGACAGTGCAG	[9]
MYC	AGCACCGAAGTCCACTTGCCT	CGCAGGAATGGGAGAAAAGACACCC	[10]
c-fos	TTTCCACGGCCTTTCCCTGTA	GTGTCCTAATCTCGTGAGCATTTCG	[7]

Supplementary References

1 Subramanian A, Tamayo P, Mootha VK, Mukherjee S, Ebert BL, Gillette MA *et al*. Gene set enrichment analysis: a knowledge-based approach for interpreting genome-wide expression profiles. *Proceedings of the National Academy of Sciences of the United States of America* 2005; 102: 15545-15550.

2 Ling H, Spizzo R, Atlasi Y, Nicoloso M, Shimizu M, Redis RS *et al*. CCAT2, a novel noncoding RNA mapping to 8q24, underlies metastatic progression and chromosomal instability in colon cancer. *Genome Res* 2013; 23: 1446-1461.

3 Yu Q, Zhou X, Xia Q, Shen J, Yan J, Zhu J *et al*. Long non-coding RNA CCAT1 that can be activated by c-Myc promotes pancreatic cancer cell proliferation and migration. *Am J Transl Res* 2016; 8: 5444-5454.

4 Alekseyenko AA, Walsh EM, Wang X, Grayson AR, Hsi PT, Kharchenko PV *et al*. The oncogenic BRD4-NUT chromatin regulator drives aberrant transcription within large topological domains. *Genes Dev* 2015; 29: 1507-1523.

5 Wang R, Liu W, Helfer CM, Bradner JE, Hornick JL, Janicki SM *et al*. Activation of SOX2 expression by BRD4-NUT oncogenic fusion drives neoplastic transformation in NUT midline carcinoma. *Cancer Res* 2014; 74: 3332-3343.

6 Alam H, Sehgal L, Kundu ST, Dalal SN, Vaidya MM. Novel function of keratins 5 and 14 in proliferation and differentiation of stratified epithelial cells. *Mol Biol Cell* 2011; 22: 4068-4078.

7 Yan J, Diaz J, Jiao J, Wang R, You J. Perturbation of BRD4 protein function by BRD4-NUT protein abrogates cellular differentiation in NUT midline carcinoma. *J Biol Chem* 2011; 286: 27663-27675.

8 Sauer M, Schuldner M, Hoffmann N, Cetintas A, Reiners KS, Shatnyeva O *et al*. CBP/p300 acetyltransferases regulate the expression of NKG2D ligands on tumor cells. *Oncogene* 2017; 36: 933-941.

9 Nagarajan S, Bedi U, Budida A, Hamdan FH, Mishra VK, Najafova Z *et al*. BRD4 promotes p63 and GRHL3 expression downstream of FOXO in mammary epithelial cells. *Nucleic Acids Res* 2017; 45: 3130-3145.

10 Grayson AR, Walsh EM, Cameron MJ, Godec J, Ashworth T, Ambrose JM *et al*. MYC, a downstream target of BRD-NUT, is necessary and sufficient for the blockade of differentiation in NUT midline carcinoma. *Oncogene* 2014; 33: 1736-1742.

Supplementary Figure Legends

Supplementary Fig. 1. Categories of cellular targets of the collected probes.

Supplementary Fig. 2. (A) Immunofluoresence detection of H3K27ac, BRD4-NUT and BRD4 wt proteins in PER-403 and TC-797 cells incubated with 5 μM A-485 for 24 h. Scale bar = 10 μm. (B) Immunoblot analysis of BRD4-NUT and BRD4 wt in HCC2429 cells incubated with 1 μM A-485 for 72 h.

Supplementary Fig. 3. (A) Quantitative RT-PCR analysis of *MYC*, *CCAT1* and *TP63* genes in PER-403 and TC-797 cells incubated with 5 μM A-485 for 6 h. Data are represented as mean ± SEM from three independent experiments. ***$P \leq 0.001$. (B) Quantitative RT-PCR analysis of *p300* and *CBP* genes in HCC2429 cells transfected with p300 or CBP siRNA or combined for 48 h. Data are represented as mean ± SEM from three technical replicates. (C) Quantitative RT-PCR analysis of *MYC*, *CCAT1* and *TP63* genes in HCC2429 cells transfected with p300 and CBP siRNAs for 72 h. Data are represented as mean ± SEM from three independent experiments. **$P \leqslant 0.01$, *$P \leqslant 0.05$.

Supplementary Fig. 4. (A) Hemacolor staining of PER-403 and TC-797 cells incubated with 5 μM A-485 for 72 h. Scale bar = 50 μm. (B) Immunoblot analysis of involucrin in PER-403 and TC-797 cells incubated with 5 μM A-485 for 72 h. (C) Quantitative RT-PCR analysis of squamous tissue genes (*KRT10*, *KRT14* and *TGM1*) and *c-fos* in PER-403 and TC-797 cells incubated with 5 μM A-485 for 72 h. Data are represented as mean ± SEM from three independent experiments. ***$P \leq 0.001$, **$P \leq 0.01$, *$P \leq 0.05$. (D) Quantitative RT-PCR analysis of squamous tissue genes (*KRT10*, *KRT14* and *TGM1*) and *c-fos* in HCC2429 cells transfected with p300 and CBP siRNAs for 5 days. Data are represented as mean ± SEM from three independent experiments. **$P \leq 0.01$, *$P \leq 0.05$; n.s., not significant.

Supplementary Fig. 5. Chromatin immunoprecipitation (ChIP) analysis of H3K27ac and BRD4-NUT at the *c-fos* promoter region in HCC2429 cells incubated with 1 μM A-485 or DMSO for 3 days. Chromatin was precipitated with normal rabbit IgG, H3K27ac and NUT antibodies. Precipitated chromatin was analyzed using qPCR and presented as fold enrichment to IgG control. Data are represented as mean ± SEM from four independent experiments. ***$P \leq 0.001$, *$P \leq 0.05$.

Supplementary Fig. 6. Combination response to A-485 and JQ1 for non-NMC cell line Patu8988S. CellTiterGlo cell viability assay was performed to measure cell viabilities of all the indicated dose combinations for 72 h. Synergy effects were evaluated using SynergyFinder (https://synergyfinder.fimm.fi). The ZIP synergy score is averaged over all the dose combination cells.

Supplementary Fig. 7. (A) Quantification of colony formation assay for HCC2429 cells incubated with 50 nM JQ1 and 250 nM A-485 alone or combined for 72 h. Cell confluence is normalized to DMSO-treated sample. Data are represented as mean ± SEM from three independent experiments. $^{**}P \leq 0.01$; n.s., not significant. (B) Hemacolor staining of HCC2429 cells incubated with 50 nM JQ1 and 250 nM A-485 alone or combined for 5 days. Scale bar = 10 µm.

Supplementary Fig. 1

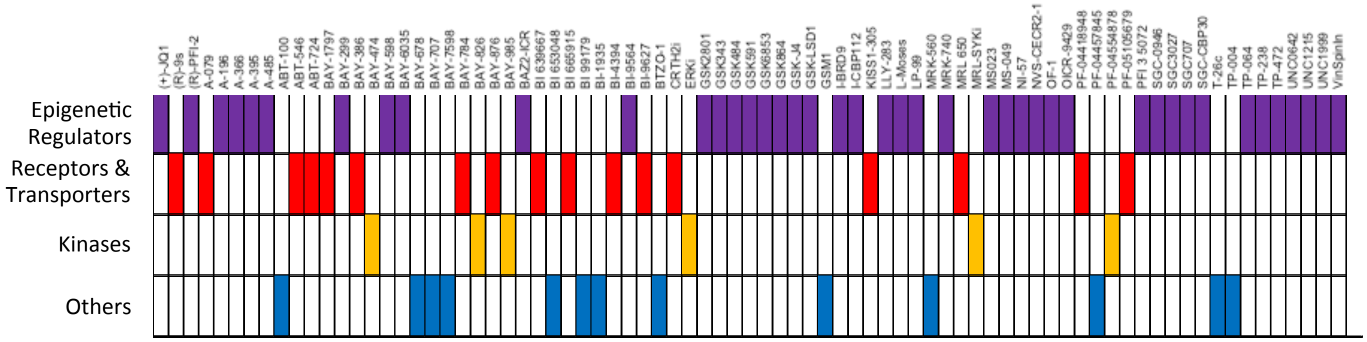

Supplementary Fig. 2.

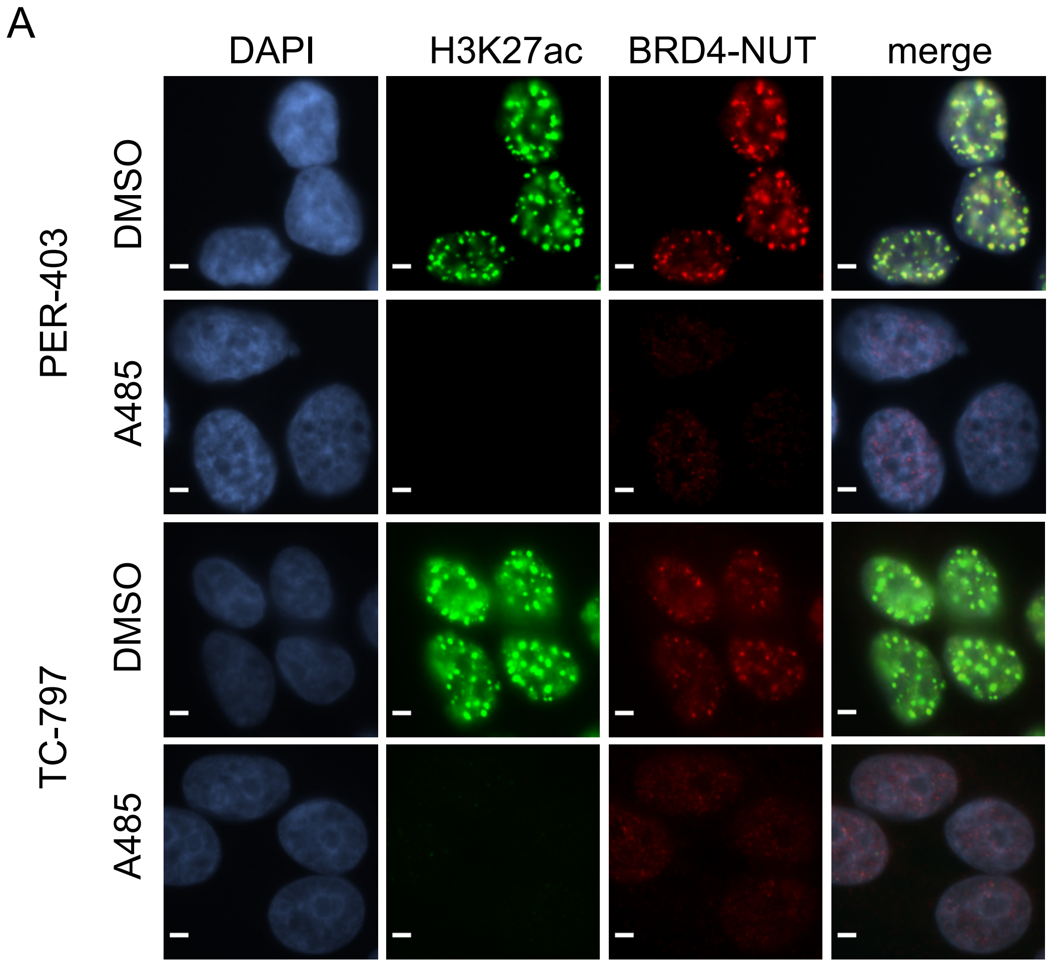

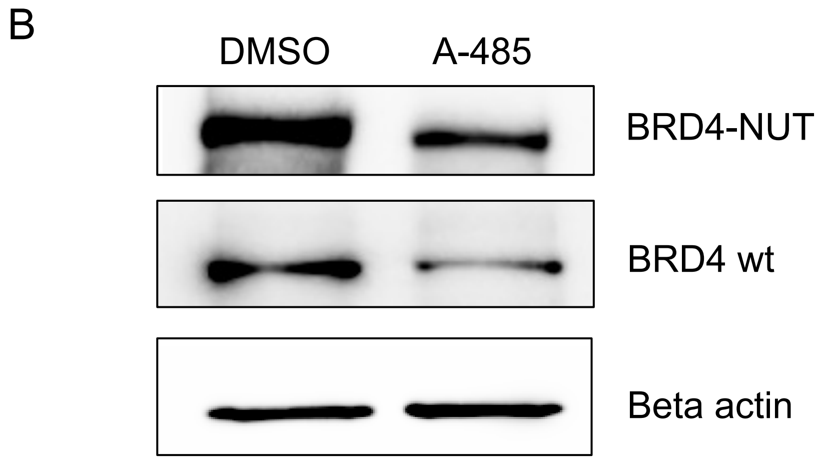

Supplementary Fig. 3

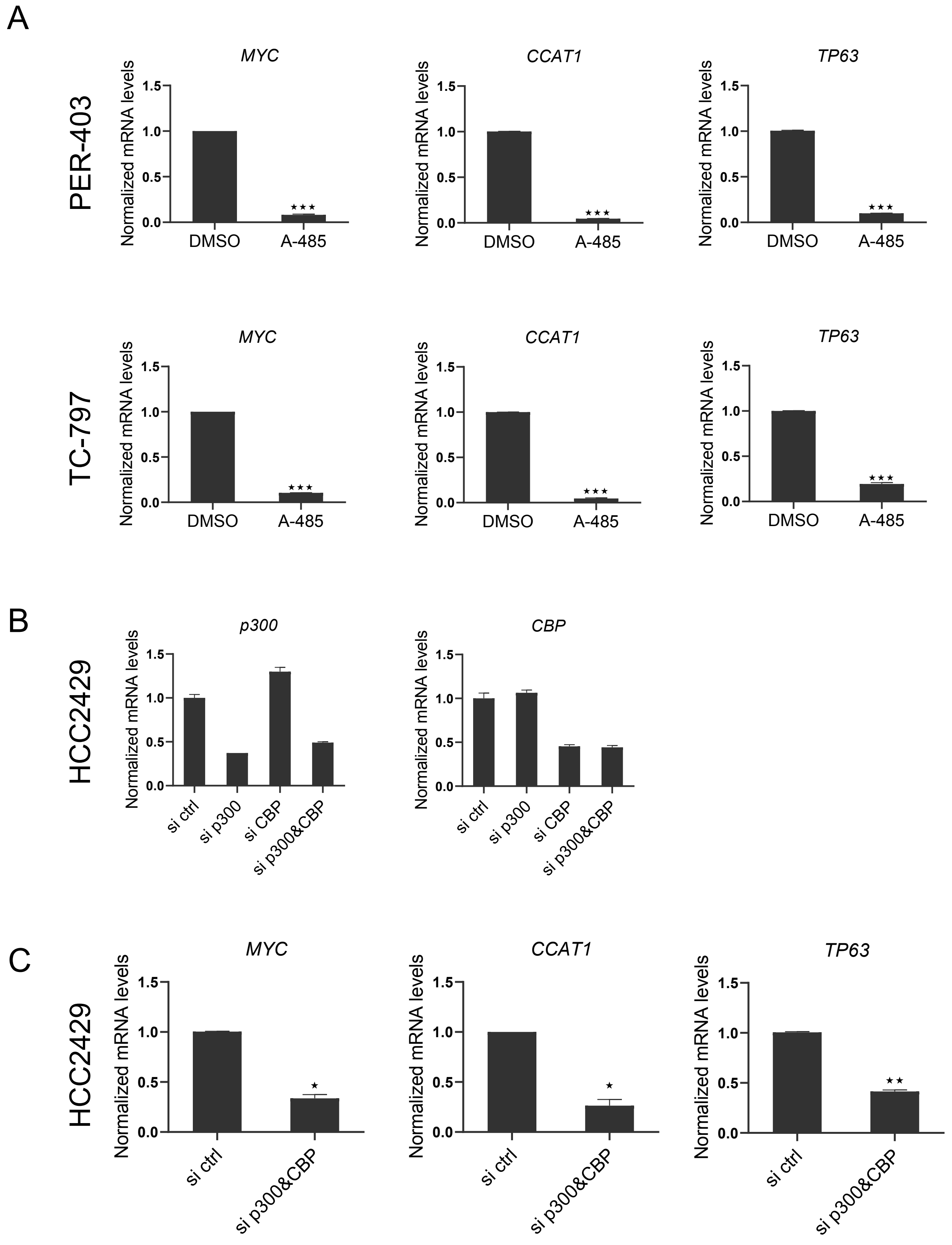

Supplementary Fig. 4

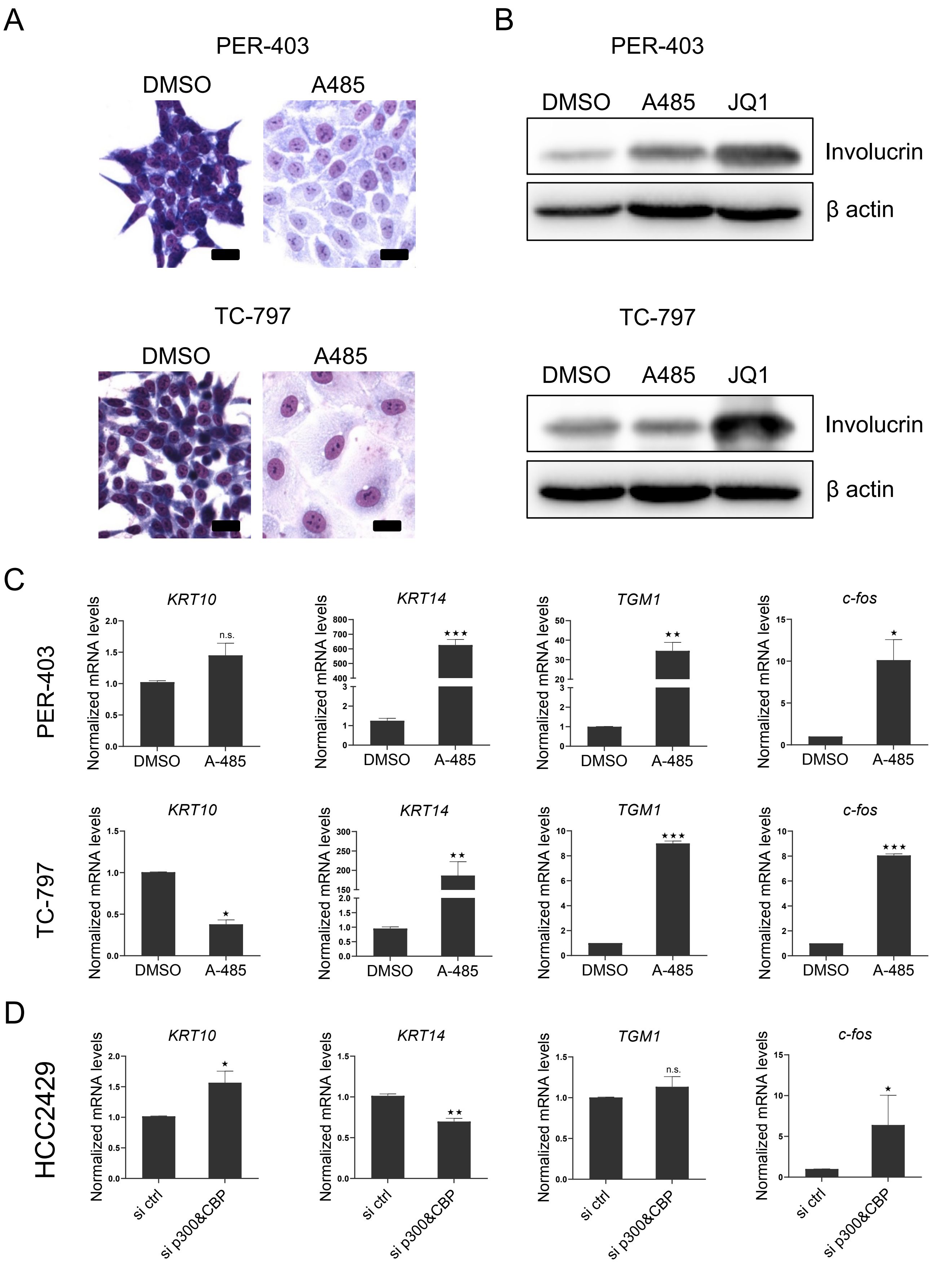

Supplementary Fig. 5

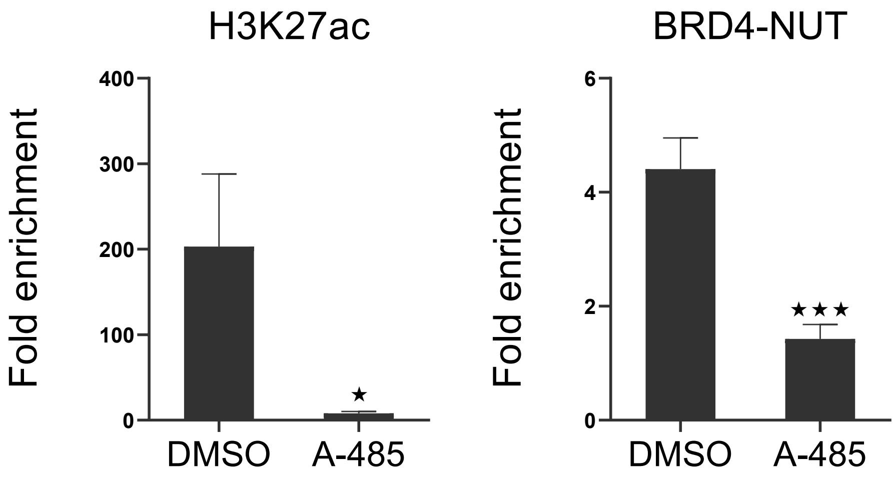

Supplementary Fig. 6

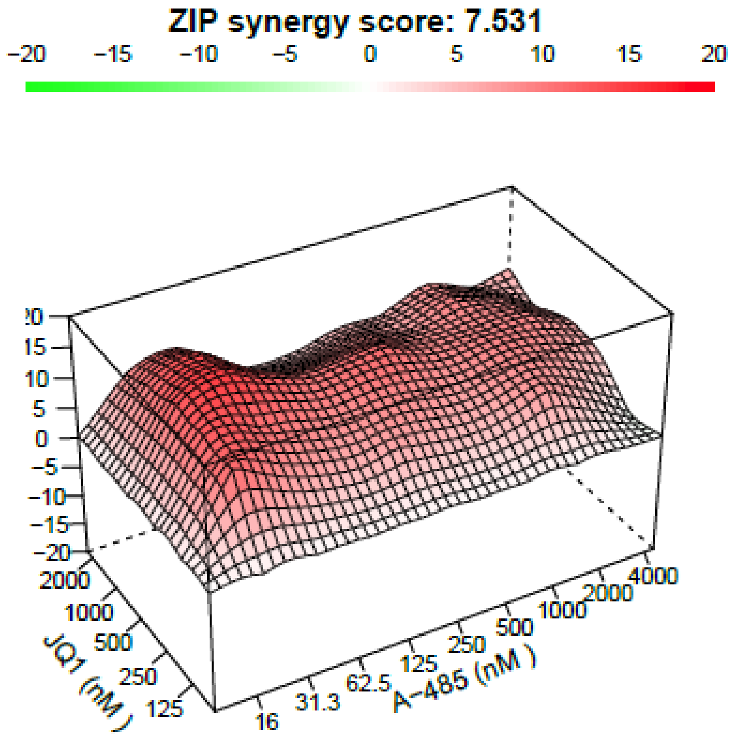

Supplementary Fig. 7

A

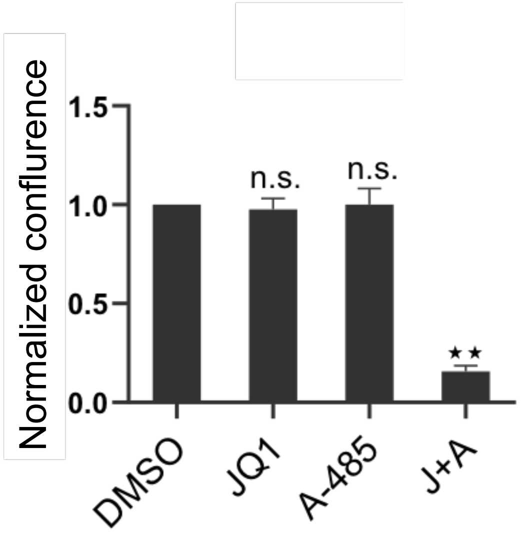

B

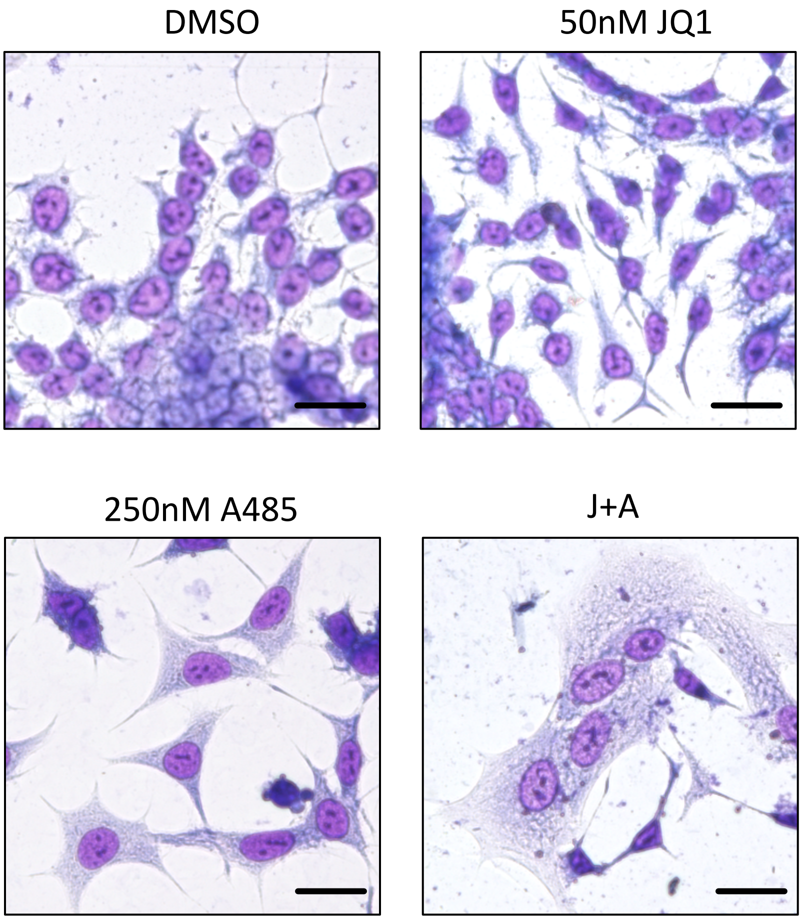

Supplement: Supplementary file 1 — Supplementary information [file 41388_2020_1301_MOESM1_ESM.pdf]
